# Activation of peripheral TRPM8 mitigates ischemic stroke by topically applied menthol

**DOI:** 10.1186/s12974-022-02553-4

**Published:** 2022-07-27

**Authors:** Shiang-Suo Huang, Hsing-Hui Su, Szu-Yu Chien, Hsin-Yi Chung, Sih-Ting Luo, Yu-Ting Chu, Yi-Hsin Wang, Iona J. MacDonald, Hsun-Hua Lee, Yi-Hung Chen

**Affiliations:** 1 Department of Pharmacology, Chung Shan Medical University, Taichung, 40201 Taiwan; 2 School of Medicine, Institute of Medicine, Chung Shan Medical University, Taichung, 40201 Taiwan; 3 Department of Pharmacy, Chung Shan Medical University Hospital, Taichung, 40201 Taiwan; 4 Graduate Institute of Acupuncture Science, China Medical University, Taichung, 40402 Taiwan; 5 Department of Neurology, Shuang Ho Hospital, Taipei Medical University, New Taipei City, 23561 Taiwan; 6 Dizziness and Balance Disorder Center, Shuang Ho Hospital, Taipei Medical University, New Taipei City, 23561 Taiwan; 7 Department of Neurology, School of Medicine, College of Medicine, Taipei Medical University, Taipei, 11031 Taiwan; 8 Department of Neurology, Taipei Medical University Hospital, Taipei Medical University, Taipei, 11031 Taiwan; 9 Chinese Medicine Research Center, China Medical University, Taichung, 40402 Taiwan; 10 Department of Computer Science and Information Engineering, Asia University, Wufeng, Taichung, 41354 Taiwan

**Keywords:** Stroke, Acute ischemic stroke, Neuroprotection, TRPM8, Menthol, Topical

## Abstract

**Background:**

No reports exist as to neuroprotective effects associated with topical activation of transient receptor potential melastatin 8 (TRPM8), a noted cold receptor. In the present study, we identified whether activating peripheral TRPM8 can be an adjuvant therapy for ischemic stroke.

**Methods:**

Menthol, an agonist of TRPM8, was applied orally or topically to all paws or back of the mouse after middle cerebral artery occlusion (MCAO). We used *Trpm8* gene knockout (*Trpm8*^*−/−*^) mice or TRPM8 antagonist and lidocaine to validate the roles of TRPM8 and peripheral nerve conduction in menthol against ischemic stroke.

**Results:**

Application of menthol 16% to paw derma attenuated infarct volumes and ameliorated sensorimotor deficits in stroke mice induced by MCAO. The benefits of topically applied menthol were associated with reductions in oxidative stress, neuroinflammation and infiltration of monocytes and macrophages in ischemic brains. Antagonizing TRPM8 or *Trpm8* knockout dulls the neuroprotective effects of topically application of menthol against MCAO. Immunohistochemistry analyses revealed significantly higher TRPM8 expression in skin tissue samples obtained from the paws compared with skin from the backs, which was reflected by significantly smaller infarct lesion volumes and better sensorimotor function in mice treated with menthol on the paws compared with the back. Blocking conduction of peripheral nerve in the four paws reversed the neuroprotective effects of topical menthol administrated to paws. On the other hand, oral menthol dosing did not assist with recovery from MCAO in our study.

**Conclusion:**

Our results suggested that activation of peripheral TRPM8 expressed in the derma tissue of limbs with sufficient concentration of menthol is beneficial to stroke recovery. Topical application of menthol on hands and feet could be a novel and simple-to-use therapeutic strategy for stroke patients.

**Supplementary Information:**

The online version contains supplementary material available at 10.1186/s12974-022-02553-4.

## Background

Ischemic stroke is associated with significant morbidity including physical dependence, cognitive decline, depression and seizures [1]. Major approaches developed to treat acute ischemic stroke fall into two categories, reperfusion therapy and neuroprotection. Reperfusion can be achieved either by thrombolysis using thrombolytic reagents such as tissue plasminogen activator (tPA), or through mechanical removal of thrombi. Important disadvantages of thrombolysis are that its therapeutic benefit reduces as time increases after stroke onset and the rapid restoration of oxygen supply has been linked to deleterious effects on brain function, leading to secondary reperfusion injury and poor outcomes [2, 3]. Moreover, neuroprotective strategies that have shown promise in experimental stroke studies have failed in clinical trials. There is a huge unmet clinical need for strategies that are neuroprotective and improve the outcomes of stroke patients.

“Thermo-transient receptor potentials (TRPs)” are a family of receptors responsible to detect a wide range of temperature stimuli, four of which (TRPV1–TRPV4) respond to heat and two (TRPA1 and TRPM8) to cold. These TRPs are expressed in primary afferent nociceptors. TRPM8 is a member of the melastatin subfamily of TRP cation channels, and it is expressed in many kinds of organs, including urogenital and respiratory system, skin and nervous system [4]. As primary afferent nociceptors in the skin, the activation range of temperature values of TRPM8 is 8–26 °C for cold-sensing [5]. Mouse knockout studies have demonstrated that TRPM8 is required for cold sensation after both innocuous and noxious cold temperatures [6–8]. In addition to cold sensing, peripheral TRPM8 is also involved in cold allodynia and cooling-induced analgesia [9, 10]. In spite of pharmacological activation of peripheral TRPM8 driving neural activity to the brain [11], no reports exist as to neuroprotective effects associated with topical activation of TRPM8.

Menthol activates TRPM8 in somatosensory neurons [9, 12]. The naturally occurring compound menthol [(1R,2R,5S)-5-methyl-2-propan-2-ylcyclohexan-1-ol] is the principal constituent of the essential oil of peppermint obtained from the leaves of various *Mentha* species. Menthol is available as prescribed and over-the-counter (OTC) pharmaceutical preparations in topical analgesic, antipruritic, antiseptic and cooling formulations. Menthol is also employed in external broncholytic and secretolytic preparations [13, 14]. The US Food and Drug Administration (FDA) considers menthol to be a safe and effective topical OTC product. FDA approval has been granted for OTC external use for menthol products containing menthol concentrations of up to 16%, which have demonstrated excellent safety profiles in postmarketing data [15]. Analgesic effects induced by menthol in animal models of acute and inflammatory pain are completely abolished in TRPM8-deficient mice, supporting a crucial role of TRPM8 in menthol-induced analgesia [16]. Menthol has also shown marked analgesic effects in models of neuropathic pain and reduces itch in a mouse model [17, 18].

The present study aimed to explore whether activation of peripheral TRPM8 by administering menthol treatment may effectively limit neurological deficits following cerebral ischemia in a mouse middle cerebral artery occlusion (MCAO) model of stroke. We hypothesized that dermal application of menthol would reduce cerebral damage and improve sensorimotor function after focal cerebral ischemic injury, and we systematically investigated its neuroprotective mechanisms. We also hypothesized that the neuroprotective effects of menthol would be antagonized by pretreatment with a TRPM8 antagonist or inhibition of the peripheral nerve, and that the protective effects would be eliminated in *Trpm8* knockout (*Trpm8*^*−/−*^) mice.

## Methods

### Animals and experimental design

All animal experiments were performed in accordance with the Guide for the Care and Use of Laboratory Animals (National Institutes of Health Publication, revised 2011) under a protocol approved by the Animal Research Committee of China Medical University (CMUIACUC-2018–111) and Chung Shan Medical University (IACUC 2397). Male C57BL/6 mice (BioLasco Taiwan Co., Ltd, Taiwan) and *Trpm8*^*−/−*^ mice (strain name: B6.129P2-*Trpm8*^*tm1Jul*^/J; Jackson Laboratory, USA) weighing 20–30 g were used in our experiments. The mice were housed 5 per cage in an approved animal research facility under controlled temperature (24 ± 1 °C), humidity (55%) and lighting conditions (12 h light/dark cycle), with freely available food and tap water. They were randomly assigned to the following study groups by electronically generated lists: Sham (no MCAO or drug application); Water (MCAO; dd water applied topically to all paws); Ethanol (MCAO; ethanol applied topically to all paws); 8% ME (MCAO; 8% menthol applied topically to all paws); 16% ME (MCAO; 16% menthol applied topically to all paws); Back ME (MCAO; 16% menthol applied topically to the back); Oral ME (MCAO; menthol 200 mg/kg); AMTB, a TRPM8 antagonist, plus ME (MCAO; AMTB 4 mg/kg i.p.; 16% menthol applied topically to all paws); lidocaine plus ME (MCAO; lidocaine (2%, 10 µl) administered intradermally to all paws; 16% menthol applied topically to all paws). Mice from different conditions were housed together in order to avoid observer bias in behavioral testing. Depending on which group the mice were assigned to, all 4 paws or the back were immersed for 1 min in water, vehicle (95% EtOH), or menthol (8% or 16%) after 20 min of MCAO under anesthetic and for 15 s once daily thereafter from Day 1 to Day 6 inclusive. Menthol dissolves in ethanol, which evaporates within a few minutes from mouse skin, and we wiped off any remaining liquid from the paws before returning the mouse to its cage. The animals were not moving in the cage with wet paws. All the experiments were performed as blind tests. Before unblinding, the animals were labeled with number without the information of treatment for evaluating behavior and mechanisms.

### The MCAO model

The MCAO method was modified from previous research [19], and male mice were used for avoiding the interferences caused the cardiovascular protective effects of estrogen. The mice were anesthetized using i.p. Zoletil^®^ (50 mg/kg, Virbac, France) and Rompun^®^ (10 mg/kg, Bayer, Germany). Core body temperature was maintained during surgery at 37 ± 0.5 °C by manual adjustment of an electric heating pad in response to measured rectal temperatures. Focal ischemic infarcts were made in the right lateral cerebral cortex in the territory of the MCA. Mice were placed in a lateral position and the skin incision was made at the midpoint between the right lateral canthus and the anterior pinna. The temporal muscle was retracted and a small (2 mm diameter) craniectomy was made at the junction of the zygoma and squamosal bone using a drill (Dremel Multipro15395, Dremel Service Center, Racine, WI, USA) cooled with saline solution. The dura was opened with fine forceps using a dissecting microscope (OPMI-1, Carl Zeiss, Oberkochen, Germany) and the right MCA was exposed and coagulated using a small-vessel cauterizer (HIF-120, WEM, Ribeirao Preto, Brazil) with simultaneous occlusion of the both common carotid arteries using microaneurysm clips for 20 min to paralyze the dominant forelimb. After the clips were removed, restoration of blood flow was visualized in the arteries.

### Assessment of ischemic infarct volume

Immediately after killing, mice were transcardially perfused with cold saline, brains were removed, sliced into 1-mm coronal sections using a mouse brain matrix (JACOBOWITZ Systems, Zivic-Miller Laboratories INC, Allison Park, USA) and stained using the TTC method, as reported previously [20]. We measured the red-colored ipsilateral and contralateral hemispheres in all slices, and the difference in volume between these hemispheres was the infarction area. An example is shown in Additional file 1: Fig. S1. Infarct volume (mm^3^) was determined by multiplying the infarction area in each 1-mm slice and summing the volume of each slice for each animal.

### Rotarod testing of motor coordination and balance alterations

Mice were subjected to accelerating rotarod testing as previously described [21]. In brief, rotarod testing recorded the length of time that a mouse stays on a rotating rod (Ugo Basile S.R.L., Monvalle, Italy) with auto acceleration from 4 rotations per minute (rpm) to 40 rpm in 4 min, to obtain latency-to-fall baseline values. Three trials were performed for each mouse and the latency (in seconds) to fall times were recorded [21].

### Measurement of sensorimotor function

An adhesive sticker (cut into equal-sided squares, 4 mm in diameter) was applied to the hair-free area of both forepaws. To ensure good attachment of the stickers to the feet, the paws were wiped with a paper towel to remove oil secreted by the skin before attaching stickers. The mouse was placed back into the test cage and the time to sense and time to remove the sticker were recorded with a maximum of 120 s. All mice were tested in the same order. Each testing session involved 3 trials. As recommended by other researchers, we trained the mice for 5 consecutive days, 1 trial per day, before subjecting them to MCAO, to ensure that they reached optimal performance [22].

### Immunohistochemistry analysis

IHC staining identified TRPM8 receptors in 3-µm-thick skin sections obtained from the backs and all paws of 4 mice in each study group. These sections were fixed in 10% formalin, as described previously in a study that used anti-TRPM8 (1:100) [23]. Micromount^®^ mounting medium (Leica, Heidelberg, Germany) was used to mount specimens. Images were acquired using a microscope (Zeiss Axio Imager A2) and the area of 3,3’-diaminobenzidine (DAB) signaling was quantified. In brief, the threshold was set for the DAB-stained IHC image and the areas of DAB-stained tissue and whole tissue in the image were analyzed with ImageJ™ software (1. 51 v, NIH) [24]. The ratios of the areas of DAB-stained tissue to whole tissue were counted.

### In situ detection of apoptosis in brain

The brains were harvested and embedded in optimal cutting temperature (OCT) compound (Leica, Heidelberg, Germany) and frozen immediately, before cutting sections of 25 μm thicknesses from each tissue block. Terminal deoxynucleotidyl transferase dUTP nick-end labeling (TUNEL) evaluated apoptosis using ApopTag Plus Fluorescein In Situ Apoptosis Detection Kits (Millipore, Burlington, MA, USA). ProLong^®^ Gold Antifade Reagent (P36971; Thermo Fisher) was used to mount specimens and stain the nuclei. Three images from the peri-infarct zone were acquired in each section using a microscope (ZEISS Axio Imager A2) and the numbers of cells were counted with ImageJ™ software (1. 51 v, NIH), as previously described [25, 26]. We used specific criteria for counting TUNEL^+^ cells, including nuclei-like size and shape, as well as fluorescent signal colocalization between green (TUNEL) and blue (nucleus) channels. The same criteria were adhered to for all images and the ratios of TUNEL^+^ cells to total nuclei were calculated by an independent observer.

### Western blot analysis

The experimental procedure followed that used in our previous study [27]. In brief, samples obtained from tissues were homogenized in a protein extraction buffer. Protein concentration was determined using the BCA protein assay kit (Pierce). Protein at 20–40 μg was separated by sodium dodecyl sulfate–polyacrylamide gel electrophoresis (SDS-PAGE) using an 8–15% resolving gel under reducing conditions and the gel was transferred to polyvinylidene difluoride (PVDF) membranes (GE Healthcare Life Sciences). After undergoing blocking with 1% BSA diluted in Tris-buffered saline (TBS) for 1 h at room temperature, the membranes were incubated overnight at 4 °C with primary antibody diluted in 0.1% Tween 20 in 20 mM Tris and 150 mM NaCl. The membranes were probed with β-actin (1:10,000) as the internal control. The blots were incubated for 1 h at room temperature with a horseradish peroxidase-conjugated secondary antibody (1:10,000; Santa Cruz Biotechnology). Protein bands were detected using Immobilon Western Chemiluminescent HRP Substrate (Millipore) and estimated using Image Analysis Program Labwork 4.5 (UVP, Inc., Upland, CA, USA).

### Immunofluorescence staining

Immunofluorescence confocal microscopy was performed according to the method described in our previous study, with some modifications [27]. In this study, GFAP as the astrocytosis marker, and the microgliosis were identified by Iba-1. At 48 h after MCAO, sections from infarcted brain were blocked and suspended in phosphate buffered saline (PBS) containing 5% donkey serum and 0.1% Triton X-100 before being treated overnight with GFAP (1:200) and Iba1(1:200) antibodies. The sections were then incubated with Alexa Fluor 488-conjugated goat anti-rabbit secondary antibody (1:500, A-11008; Thermo Fisher) for 1 h, then mounted on slides with the coverslip sealed with ProLong™ Diamond Antifade Mountant with DAPI (P36971; Thermo Fisher). Images were taken with a confocal microscope (model SP8 TCS; Leica, Heidelberg, Germany). To quantify the levels of GFAP and Iba1, the corrected total cell fluorescence (CTCF) was analyzed. The cells of interest were selected using ImageJ™ software tools (1. 51 v, NIH), which analyzed the areas, integrated intensities and mean gray values. The CTCF was calculated with this formula: CTCF = integrated density − (area of selected cell × mean fluorescence of background readings) [28, 29].

### Leukocyte and microglia isolation from mouse brains and flow cytometry analysis

Microglia and monocytes/macrophages in the infarcted brains were analyzed using flow cytometry [30]. At 24 h after MCAO, the mice were killed with an overdose of isoflurane and then transcardially perfused with PBS. The brains were rapidly removed and then dissociated with a tissue grinder in ice-cold Accutase (Innovative Cell Technologies, Inc., San Diego, CA, USA) to obtain the single-cell suspension. After centrifugation, the cell pellet was resuspended in a 37% PBS-Percoll (Cytiva, Marlborough, MA, USA) and set up the Percoll density gradient to 30–37–70% (from top to bottom). Centrifuge gradient for 40 min at 300×*g* at room temperature without brakes. The tube will appear stratified. The cells layered at the 37–70% density gradient interface are the mononucleated immune cells (microglia and macrophage subpopulations) [31]. Anti-mouse CD16/32 antibody (TruStain FcX™ PLUS, Biolegend, San Diego, CA, USA) was used to block the Fc receptors, and then the cells were incubated with fluorescent-conjugated CD11b (1:200; FITC anti-mouse/human CD11b Antibody, Biolegend) and CD45 antibodies (1:100; PerCP anti-mouse CD45 Antibody, Biolegend) for 30 min at 4 °C. Cells were resuspended in staining buffer (Biolegend) for analysis using the flow cytometer (BD FACSCanto, BD Biosciences, San Jose, CA, USA) [30]. Based on the limitation of this technique resides, the efficiency of cell isolation was affected by the processing (tissue dissection, disruption procedure, and density gradient stratified) and provides an inconstant cell yield. We gated the population 1 (P1) area of target cells according to particle size and granularity, and we excluded cell debris and other irrelevant particles as much as possible. Since the cell yield and quality by density gradient isolation were not identical between sample batches, we used two brains (additional mice) to adjust the measurement parameters and frame the area of P1. CD45^high^/CD11b^+^ and CD45^low^/CD11b^+^ populations were regarded as infiltrating monocytes/macrophages and resident microglia in the brain, respectively [30–32]. To avoid data variability due to this approach, the number of specific fluorescent stained cells in the quadrants was normalized to the percentage of P1, instead of the absolute value.

### Drugs and antibody

Menthol (Alfa Aesar); AMTB and lidocaine (TOCRIS); anti-TRPM8 (Abcam); anti-GFAP, anti-PARP, and anti-caspase-3 (Cell Signaling); Iba1 (Novus); anti-CD11b and anti-β-actin (Genetex); FITC-anti-CD11b, and PerCP-anti-CD45 antibodies (Biolegend).

### Statistical analysis

The results are presented with actual data points and expressed as the means ± standard error of the mean (S.E.M.). The result of behavior tests and infarction size for ME treatment in wild type or *Trpm8* knockout mice were assessed using two-way or three-way repeated measures analysis of variance (ANOVA), and the Holm–Sidak test was used for post hoc pairwise multiple comparisons where necessary. Other results were assessed using one-way ANOVA followed by the Duncan’s test or the Kruskal–Wallis test followed by the Dunn’s method, according to the Shapiro–Wilk normality test. The Student’s *t*-test or Mann–Whitney *U* test was used only to compare between two groups, according to normality test. *p* < 0.05 was considered significant.

## Results

**Fig. 1:**
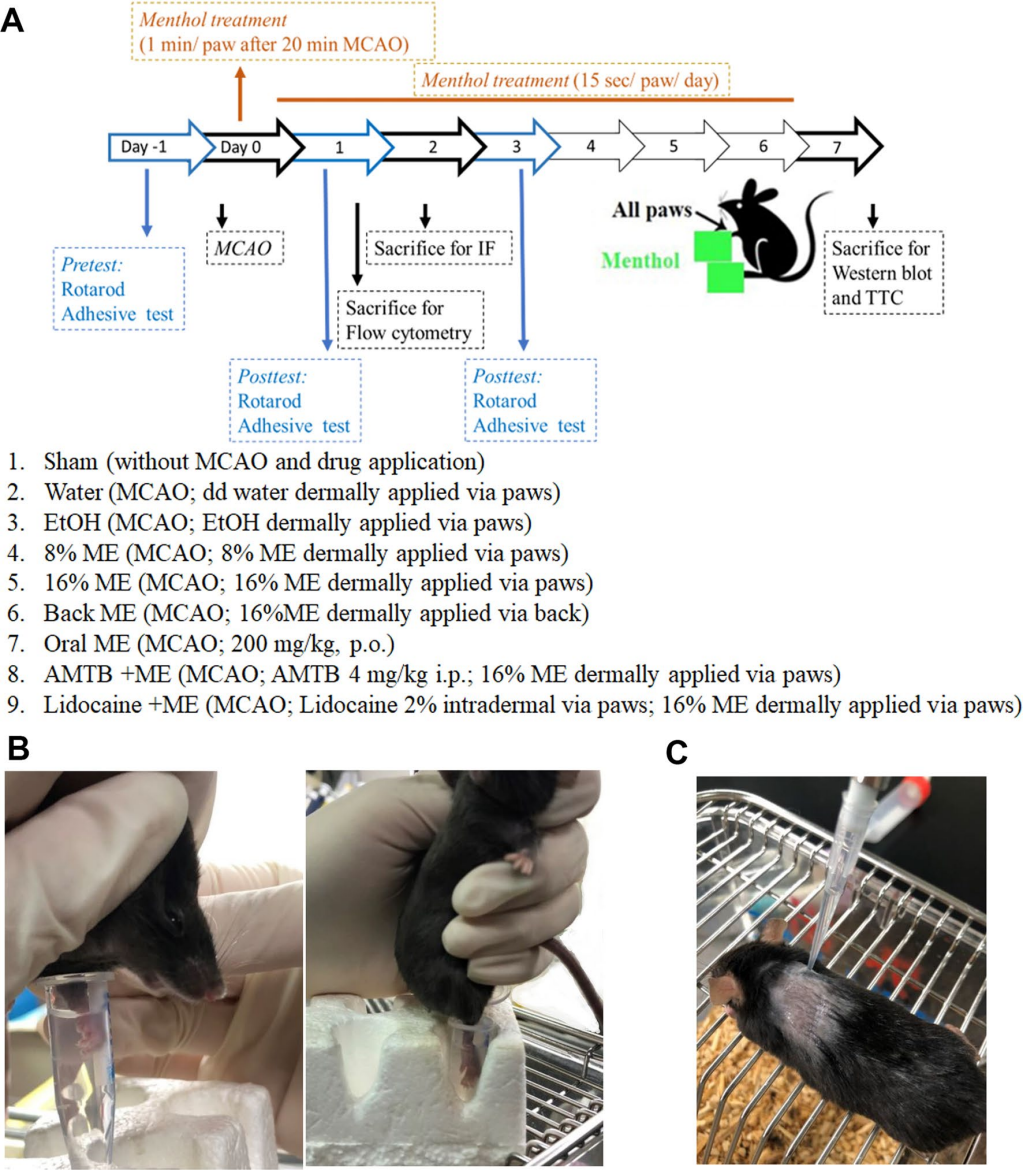
Timeline of the experimental procedure for ischemic stroke induction and menthol (ME) application. **A** ME (8% or 16% in 95% ethanol), water or ethanol  was applied topically for 1 min to each paw or to the back after 20 min of MCAO, and for 15 s once daily thereafter until Day 6. Oral treatment consisted of menthol (200 mg/kg) administered via oral gavage. The AMTB-treated group was administered a single AMTB (4 mg/kg i.p.) injection 30 min before menthol treatment. Lidocaine-treated mice received lidocaine injections (2%, 10 μL) in the wrists and ankles before topical menthol application. All mice were subjected to 20 min of MCAO and observed for 1, 2 or 7 days. Behavior testing was conducted at baseline and on Days 1, 3 and 6. **B** Front and hind paws of mice were immersed in menthol, water or ethanol. **C** 16% ME (200 µL) was applied to the skin of the back and then was wiped off after 1 min

Figure 1A illustrates the timeline of the experimental procedure for stroke induction and menthol application. Menthol (8% or 16% in 95% ethanol) was applied topically to all paws for 15 s (Fig. 1B) or to the back for 1 min (Fig. 1C) of each mouse after 20 min of middle cerebral artery occlusion (MCAO) and once daily thereafter until Day 6. Oral treatment consisted of 200 mg/kg menthol administered via gavage. Mice treated with the specific TRPM8 antagonist AMTB (*N*-(3-aminopropyl)-2-[(3-methylphenyl)methoxy]-*N*-(2-thienylmethyl)benzamide) were administered a single intraperitoneal (i.p.) AMTB (4 mg/kg) injection 30 min before menthol treatment. Lidocaine-treated mice received lidocaine injections (2%, 10 μL) in all wrists before topical menthol application. All mice were subjected to MCAO and observed for 2 or 7 days.

### Topically applied menthol reduced infarct volumes and improved sensorimotor deficits in MCAO mice

**Fig. 2:**
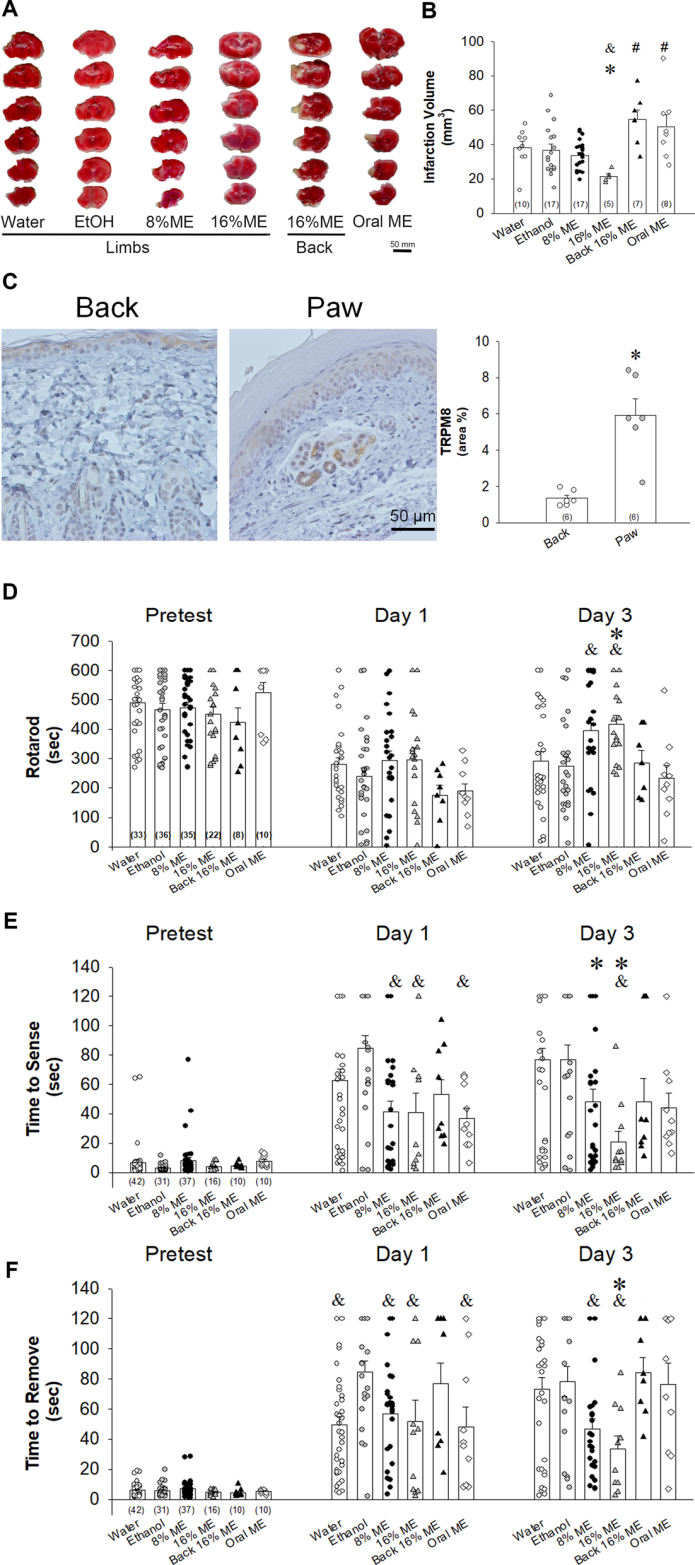
Topically applied menthol (ME) attenuates acute cerebral infarction and ameliorates ischemia-induced sensorimotor deficits. **A** TTC staining of cerebral infarction volume in the water controls, ethanol (vehicle)-treated mice, mice administered ME (8% or 16%) to the paws, mice administered ME (16%) to the back, and mice administered oral ME (200 mg/kg). Scale = 5 mm. **B** Infarct volumes (mm^3^) in ischemic brain tissue after 1 week of MCAO in the water controls,  ethanol-treated mice, and mice administered ME. **C** Representative IHC staining of TRPM8 expression in the skin from the back and paw. Scale = 50 µm. **D** Postischemic behavioral outcomes in the rotarod test. **E**, **F** Postischemic behavioral outcomes in the adhesive removal test: sense time (**E**) and latency-to-removal time (**F**). All data are expressed as the means ± S.E.M. *p < 0.05 vs water; ^&^*p* < 0.05 vs ethanol; ^#^*p* < 0.05 vs ME 16% alone. The Mann–Whitney *U* test was used only to compare levels of TRPM8 expression between skin samples from the paws and backs of 4 mice. In each bar in **B**–**F**, the number of individuals of each group was shown within parentheses, and the symbols indicate the data of mice used in each study group

When applied to the paws, menthol attenuated acute cerebral infarction and ischemia-induced sensorimotor deficits (Fig. 2). After 1 week of MCAO, 2,3,5-triphenyltetrazolium chloride (TTC)-stained tissue revealed significantly smaller infarct lesion volumes in mice treated with menthol 16% to the paws (21.5 ± 1.5 mm^3^) compared with mice in the water control (38.1 ± 3.7 mm^3^) and ethanol (vehicle) groups (36.7 ± 3.5 mm^3^) (*p* < 0.05, one-way ANOVA followed by Duncan post hoc test, Fig. 2A, B). Immunohistochemistry (IHC) staining of TRPM8 expression in skin tissue samples revealed significantly higher levels in skin obtained from the paws compared with skin from the back (4.98 ± 0.93% vs. 1.34 ± 0.19%, *p* < 0.05, one-way ANOVA followed by Duncan post hoc test, Fig. 2C). Infarct volume was only slightly reduced by menthol 8% applied to the paws and there were no improvements in infarct volume when menthol 16% was applied to the back or when menthol was given via oral gavage (200 mg/kg).

The rotarod test is proven to be a sensitive method for measuring sensorimotor function and motor coordination [33]. Topical application of menthol 8% or menthol 16% to the paws had no significant effects upon rotarod performance on Day 1 (Fig. 2D), but latency-to-fall values were significantly increased on Day 3 compared with those in ethanol-treated mice (Fig. 2D; time points: *F*_2,358_ = 67.948, *p* < 0.001; groups: *F*_5,358_ = 3.620, *p* < 0.01; time points × groups: *F*_10,358_ = 2.092, *p* < 0.05; 396.1 ± 36.9 (8%)/414.3 ± 30.2 (16%) vs. 274.9 ± 31.4 (ethanol) on Day 3, *p* < 0.05; two-way ANOVA followed by Holm–Sidak post hoc test). At the same time, latency-to-fall values were significantly prolonged by topical menthol 16% applied to the paws compared with values in the water control group (Fig. 2D; 414.3 ± 30.2 (16%) vs. 292.6 ± 32.0 (water), *p* < 0.05 on Day 3; *p* < 0.05). No such significant improvements were seen with oral menthol treatment on Day 1 and Day 3.

The adhesive removal test is widely used as a sensitive measure of sensorimotor function in mice [22] and it can identify sensorimotor deficits caused by unilateral lesions placed in distinct areas of the rat sensorimotor cortex [34]. We used the adhesive removal test in this study to determine the effects of menthol on sensorimotor deficits in MCAO mice. On Day 1 after MCAO injury, ethanol was associated with prolonged removal time compared with water treatment groups (Fig. 2F; removal: time points: *F*_2,352_ = 119.297, *p* < 0.001; groups: *F*_5,352_ = 5.740, *p* < 0.001; time points × groups: *F*_10,352_ = 3.500, *p* < 0.001; 84.8 ± 7.1 (ethanol) vs. 49.3 ± 5.8 (water), *p* < 0.01 on Day 1; two-way ANOVA followed by Holm–Sidak post hoc test). In mice administered menthol to the paws, both concentrations of menthol were associated with significantly shorter time to find and remove the adhesive pads compared with either water or ethanol treatment on Day 1 and Day 3 after MCAO injury (Fig. 2E; sense: time points: *F*_2,352_ = 64.648, *p* < 0.001; groups: *F*_5,352_ = 8.300, *p* < 0.001; time points × groups: *F*_10,352_ = 3.224, *p* < 0.001; 41.2 ± 7.3 (8%)/38.3 ± 12.3 (16%) vs. 84.8 ± 8.5 (ethanol) on Day 1, *p* < 0.01; 47.9 ± 8.6 (8%)/20.6 ± 7.6 (16%) vs. 76.8 ± 7.8 (water)/76.9 ± 10.1 (ethanol) on Day 3, *p* < 0.01; two-way ANOVA followed by Holm–Sidak post hoc test) (Fig. 2F; removal: 57.0 ± 6.0 (8%)/52.0 ± 13.9 (16%)/49.3 ± 5.8 (water) vs. 84.8 ± 7.1 (ethanol) on Day 1, *p* < 0.05; 47.0 ± 6.6 (8%)/33.4 ± 8.9 (16%) vs. 76.4 ± 7.8 (water)/78.6 ± 7.6 (ethanol) on Day 3, *p* < 0.01). Time to find and remove the adhesive pads were significantly shorten in the mice given oral menthol compared with the ethanol treatment group on Day 1, but the effects were lost on Day 3 (Fig. 2E and F; sense: 36.9 ± 6.7 (oral) vs. 84.8 ± 8.5 (ethanol) on Day 1, *p* < 0.01; removal: 48.2 ± 13.0 (oral) vs. 84.8 ± 7.1 (ethanol) on Day 1, *p* < 0.05).

### Inhibiting TRPM8 activity and peripheral nerve conduction blocked the effects of menthol on infarction volume and post-ischemic sensorimotor functions

**Fig. 3:**
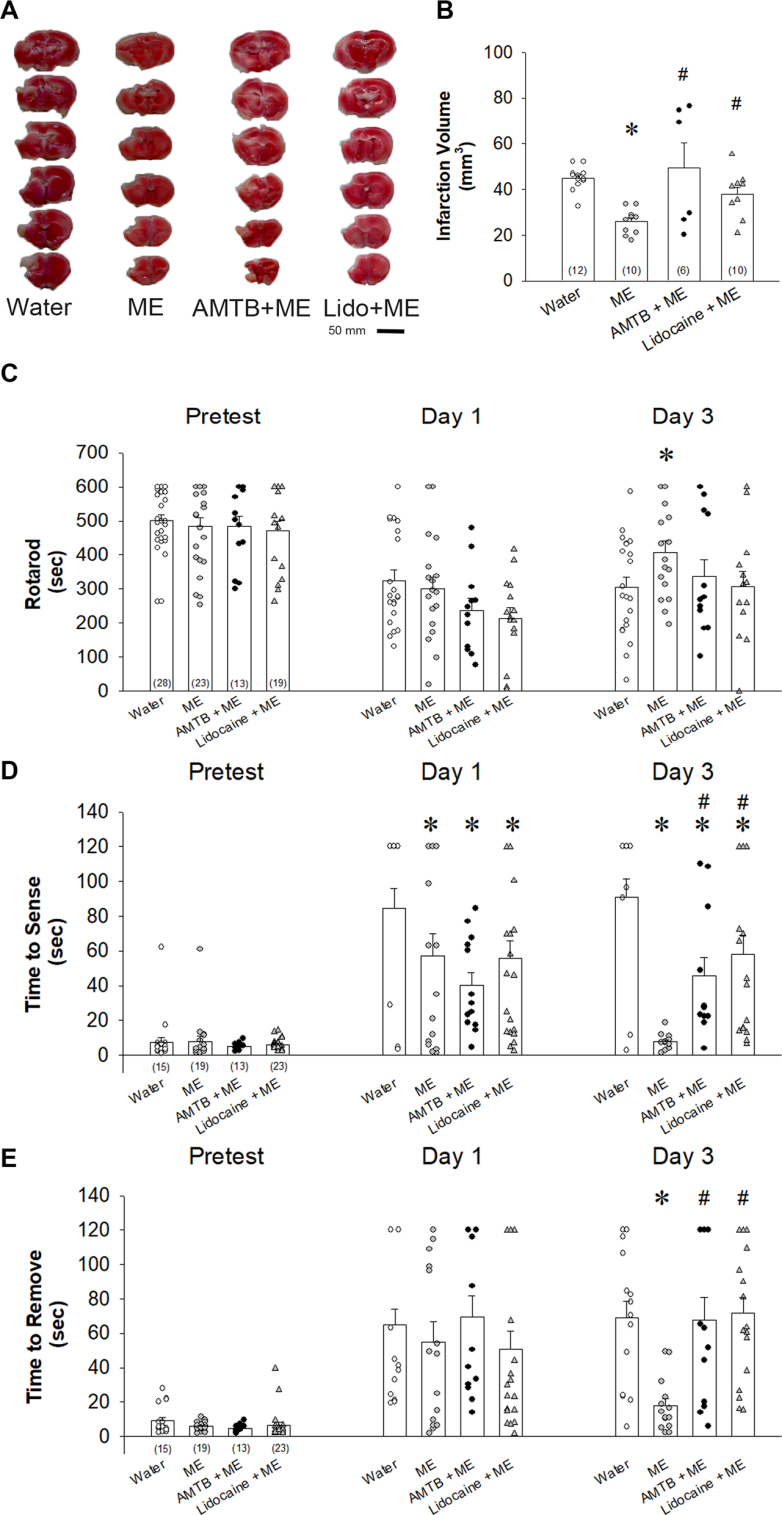
Blocking TRPM8 activation increases cerebral infarction volume and worsens sensorimotor function after MCAO in the controls, mice treated with 16% menthol (ME) alone, AMTB plus ME, or lidocaine plus ME. **A** TTC staining of cerebral infarction volume. Scale = 5 mm. **B** Infarct volumes (mm^3^) assessed in ischemic brain tissue after 1 week MCAO. **C** Rotarod performance. **D**, **E** Adhesive testing showing contact times (**D**) and latency-to-removal times (**E**). The results are shown as the means ± SEM. *p < 0.05 vs water; ^#^*p* < 0.05 vs ME alone. In each bar in **B–E**, the number of individuals of each group was shown within parentheses, and the symbols indicate the data of mice used in each study group

After 1 week of MCAO, TTC-stained infarcted tissue revealed that the effects of topically applied menthol 16% to the paws on MCAO-induced ischemic damage were substantially prevented by AMTB; lidocaine treatment also blocked the effect of menthol (Fig. 3A, B). Between-group differences in infarct sizes were significant for menthol-treated mice (25.9 ± 1.7 mm^3^) and controls (44.9 ± 1.5 mm^3^), and for mice pretreated with AMTB (49.6 ± 10.8 mm^3^) or lidocaine (37.8 ± 3.1 mm^3^) compared with those administered menthol alone (*p* < 0.05, one-way ANOVA followed by Duncan post hoc test, Fig. 3B).

Rotarod performance tended to be worse with both AMTB and lidocaine treatment compared with menthol alone; the significant between-group difference was for menthol alone but not for AMTB+menthol and lidocaine+menthol groups versus water control on Day 3 (Fig. 3C; time points: *F*_2,201_ = 48.835, *p* < 0.001; groups: *F*_3,201_ = 2.477, *p* = 0.063; time points × groups: *F*_6,201_ = 1.425, *p* = 0.196; 408.2 ± 33.3 (menthol) vs. 259.4 ± 31.4 (water) on Day 3, *p* < 0.05; two-way ANOVA followed by Holm–Sidak post hoc test). On Day 1 after MCAO injury, treatment with menthol on paws improved time to find the adhesive pads in spite of pretreatment with AMTB or lidocaine (Fig. 3D, time points: *F*_2,174_ = 48.770, *p* < 0.001; groups: *F*_3,174_ = 12.639, *p* < 0.001; time points × groups: *F*_6,174_ = 5.292, *p* < 0.001; 84.5 ± 11.6 (water) vs. 57.1 ± 12.8 (menthol)/40.2 ± 7.4 (AMTB)/56.0 ± 9.8 (lidocaine) on Day 1, *p* < 0.05; two-way ANOVA followed by Holm–Sidak post hoc test). Results of adhesive testing revealed that pretreatment with AMTB or lidocaine prolonged the time to sense the adhesive patches compared with menthol monotherapy; between-group differences were significant on Day 3 (Fig. 3D; 7.7 ± 1.3 (menthol) vs. 45.8 ± 10.2 (AMTB)/58.0 ± 11.2 (lidocaine)/90.8 ± 10.8 (water), *p* < 0.01). As for adhesive patch removal time, pretreatment with either AMTB or lidocaine significantly prolonged time compared with menthol alone (Fig. 3E; time points: *F*_2,174_ = 50.702, *p* < 0.001; groups: *F*_3,174_ = 4.222, *p* < 0.01; time points × groups: *F*_6,174_ = 3.014, *p* < 0.01; 18.0 ± 4.2 (menthol) vs. 67.8 ± 12.9 (AMTB)/71.6 ± 9.43 (lidocaine)/71.8 ± 8.75 (water) on Day 3, *p* < 0.001; two-way ANOVA followed by Holm–Sidak post hoc test).

### Topically applied menthol to paws did not improve apoptosis but did reduce oxidative damage after focal cerebral I/R injury

**Fig. 4:**
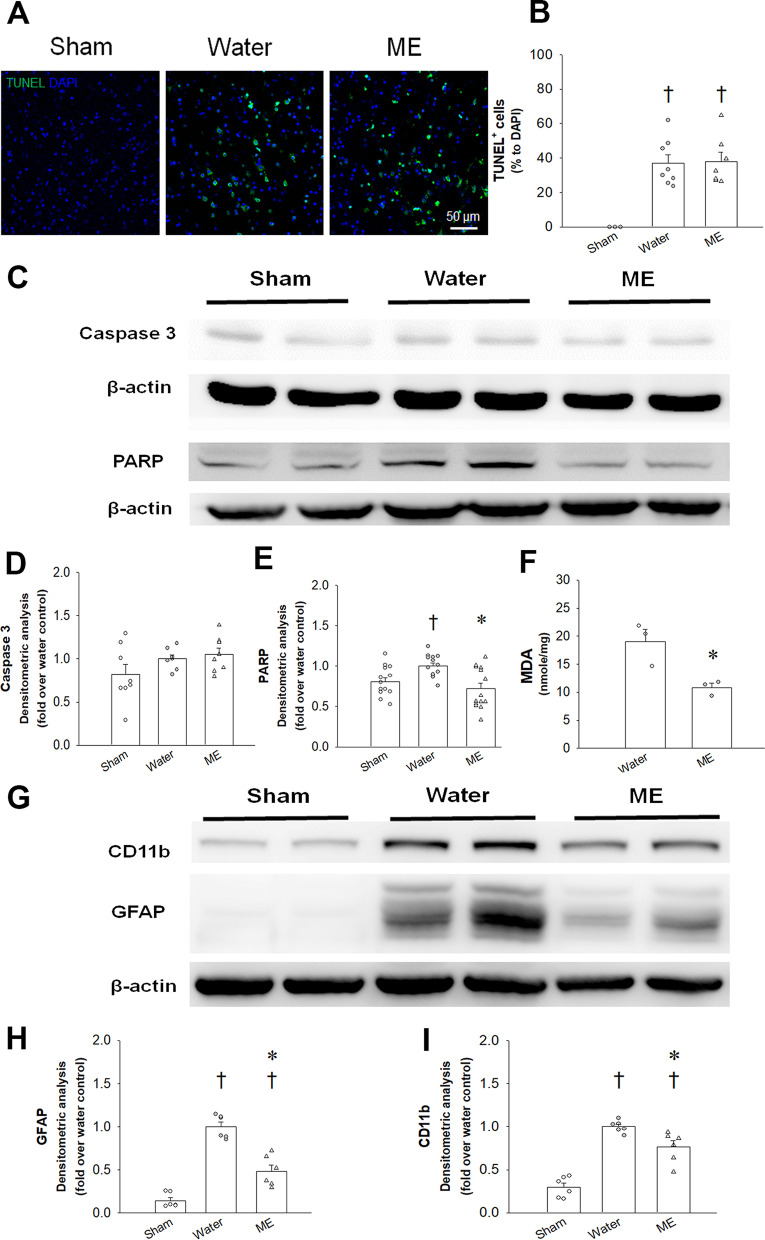
Topically applied menthol (ME) did not influence apoptosis, but suppressed markers of microglia and astrocyte activation and oxidative stress in ipsilateral brain. **A–B** Representative images and group analysis show that the expression of TUNEL (green color) was significantly increased among the water controls and mice in the ME-treated group compared with the sham-operated group (*n* = 6–8) on Day 2 after MCAO. **C** Western blot results display the expression of caspase-3 and PARP on Day 7 after ischemic stroke. β-Actin was used as the internal control. **D–E** Relative protein levels of caspase-3 (**D**) and PARP (**E**) were quantified after normalization to β-actin (*n* = 8–14). **F** Cerebral oxidative stress markers were evaluated by MDA content (*n* = 3) on Day 2 after ischemic stroke. **G** Western blot results display CD11b and GFAP expression. β-Actin was used as the internal control. **H–I** Relative protein levels of CD11b (**H**) and GFAP (**I**) on Day 7 after ischemic stroke were quantified after normalization to β-actin (*n* = 6). The results are shown as the means ± S.E.M. ^†^*p* < 0.05 vs sham; *p < 0.05 vs water

Focal cerebral ischemic injury is associated with apoptosis [35]. In this study, the TUNEL assay revealed a significant increase in apoptotic DNA fragmentation after MCAO-induced focal cerebral ischemic injury, with no reduction after topical menthol treatment compared with water treatment (Fig. 4A and B; *F*_2,18_ = 21.935, *p* < 0.001; 0.00 ± 0.00 (sham) vs. 37.05 ± 4.78 (water)/37.98 ± 5.33 (menthol) % to DAPI, *p* < 0.05; one-way ANOVA followed by Duncan post hoc test). Similarly, on Day 7 after MCAO, there were no significant between-group differences among the sham-, water- and menthol-treated groups in levels of caspase-3, a crucial mediator of apoptosis (Fig. 4C, D). Poly (ADP-ribose) polymerase (PARP), a DNA repair enzyme that is typically activated by single-strand breaks associated with oxidative stress, was increased on Day 7 after MCAO (Fig. 4C and E; *F*_2,39_ = 7.587, *p* < 0.01; 0.81 ± 0.05 vs. 1.00 ± 0.04 fold of water, *p* < 0.05; one-way ANOVA followed by Duncan post hoc test). Interestingly, topically applied menthol treatment significantly decreased PARP expression in the ipsilateral hemisphere (Fig. 4E; 1.00 ± 0.04 vs. 0.72 ± 0.07 fold of water, *p* < 0.01). Moreover, levels of malondialdehyde (MDA), an oxidative stress marker, were reduced by topical menthol application on Day 2 after MCAO (Fig. 4F; 18.98 ± 2.20 (water) vs. 10.83 ± 0.76 (menthol) nmol/mg, *p* < 0.05; *t*-test).

### Topically applied menthol to paws reduced post-stroke levels of astrocytosis and microgliosis expression

**Fig. 5:**
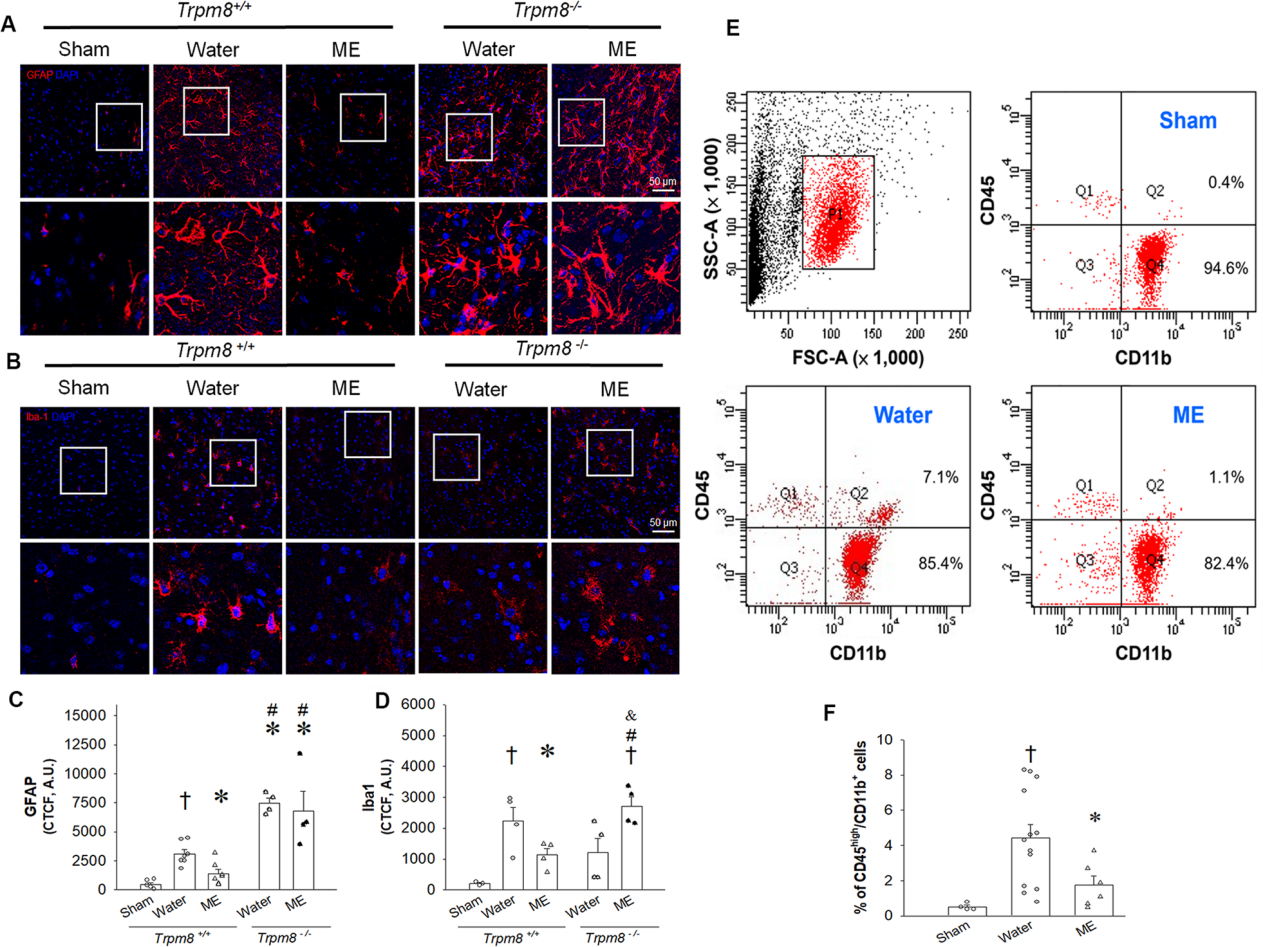
Topically applied menthol (ME) to paws mitigates MCAO-induced astrocytosis and microgliosis. **A**, **B** Representative immunofluorescent photomicrographs of GFAP (**A**) and Iba1 (**B**) expression (red color), respectively, in the peri-infarct zone of brain segments from sham-operated mice, water- and menthol-treated *Trpm8*^+*/*+^ mice and *Trpm8*^*−/−*^ mice on Day 2 following MCAO. The inset in low magnification is shown with a high magnification view in the below rows. DAPI staining is presented in blue. **C**, **D** Quantitative analysis of GFAP-positive astrogliosis and Iba1-positive microgliosis. ^†^*p* < 0.05 vs sham; *p < 0.05 vs water-treated *Trpm8*^+*/*+^ mice; ^#^*p* < 0.05 vs menthol-treated *Trpm8*^+*/*+^ mice; ^&^*p* < 0.05 vs water-treated *Trpm8*^*−/−*^ mice. **E** Density-plot graphs of FACS-sorted cells from sham-operated mice, water- and menthol-treated *Trpm8*^+/+^ mice on Day 1 following MCAO. Target cells (population 1, P1) are gated according to particle size and granularity and excluding cell debris and other irrelevant particles as much as possible. Q2 cell population, CD45^high^/CD11^+^ cells (monocytes/macrophages); Q4 cell population, CD45^low^/CD11^+^ cells (microglia). **F** Quantitative analysis of CD45^high^/CD11^+^ cell percentage in the brains. All data are expressed as the means ± S.E.M. In each bar, the symbols indicate the data of mice (*n* = 4–13) used in each study group. The scale bars represent 50 µm at 200X magnification

Levels of glial fibrillary acidic protein (GFAP) protein expression at 7 days after ischemic injury were increased at the lesion site due to astrocytic hypertrophy and were reduced by topical menthol application on the paws in wild-type (*Trpm8*^+*/*+^) mice (Fig. 4G and H; *F*_2,15_ = 58.661, *p* < 0.001; 1.00 ± 0.05 (water) vs. 0.15 ± 0.03 (sham)/0.48 ± 0.07 (menthol) fold of water, *p* < 0.05; one-way ANOVA followed by Duncan post hoc test). At 48 h after MCAO, immunofluorescence staining of brain tissue sections revealed significantly downregulated levels of GFAP-positive astrogliosis (red color) in the peri-infarct zones of menthol-treated mice compared with water-treated *Trpm8*^+*/*+^ mice (Fig. 5A and C; *F*_4,23_ = 22.239, *p* < 0.001; 3094.9 ± 370.1 (water) vs. 449.1 ± 142.7 (sham)/1400.5 ± 366.6 (menthol) corrected total cell fluorescence (CTCF), *p* < 0.05; one-way ANOVA followed by Duncan post hoc test). The CD11 protein is a marker of macrophages, monocytes and microglia, and is increased after stroke [36]. On Day 7 after MCAO, CD11b expression was markedly increased in the water treatment group and was significantly reduced by topical menthol application (Fig. 4G and I; *F*_2,15_ = 46.048, *p* < 0.001; 1.00 ± 0.03 (water) vs. 0.3 ± 0.05 (sham)/0.77 ± 0.07 (menthol) fold of water, *p* < 0.05; one-way ANOVA followed by Duncan post hoc test). At 48 h after MCAO, immunofluorescence staining showed that ionized calcium-binding adapter molecule 1 (Iba1)-positive microgliosis (red color) was significantly downregulated in the peri-infarct zone of *Trpm8*^+*/*+^ mice in the menthol-treated group compared with water-treated mice (Fig. 5B and D; *F*_4,14_ = 7.271, *p* < 0.01; 2235.7 ± 444.9 (water) vs. 208.4 ± 31.3 (sham)/1130.2 ± 213.7 (menthol) CTCF, *p* < 0.05; one-way ANOVA followed by Duncan post hoc test). In contrast, menthol treatment failed to prevent post-stroke astrogliosis and microgliosis in *Trpm8*^*−/−*^ mice. Images taken at high magnification show marked increases in GFAP-positive astrocyte expression in the water- and menthol-treated *Trpm8*^*−/−*^ mice compared with water-treated *Trpm8*^+/+ ^mice (Fig. 5A and C; 3094.9 ± 37.1 (water-*Trpm8*^+*/*+^) vs. 7450.9 ± 445.4 (water-*Trpm8*^*−/−*^)/6769.6 ± 1709.2 (menthol-*Trpm8*^*−/−*^) CTCF, *p* < 0.05). In addition, ischemic injury did not increase Iba1 expression in water-treated *Trpm8*^*−/−*^ mice compared with *Trpm8*^+*/*+^ mice, whereas Iba1-positive microglia were significantly increased in menthol-treated *Trpm8*^*−/−*^ mice compared with menthol-treated *Trpm8*^+*/*+^ animals (Fig. 5B and D; 2515.3 ± 305.4 (menthol-*Trpm8*^*−/−*^) vs. 1130.2 ± 213.7 (menthol-*Trpm8*^+*/*+^) CTCF, *p* < 0.05). Quantifications of the CTCF of GFAP and Iba1 immunoreactivity are depicted in Fig. 5C, D.

### Topically applied menthol to paws reduced monocyte infiltration into the infarcted brain after focal cerebral I/R injury

At 24 h after MCAO, leukocytes were isolated from the whole brains, and the percentages of CD45^high^/CD11b^+^ cells (Q2 population, infiltrating monocytes/macrophages) and CD45^low^/CD11b^+^ cells (Q4 population, brain-resident microglia) [37] were quantified by flow cytometry (Fig. 5E). Percentages of CD45^high^/CD11b^+^ cells in the brain were significantly higher in the water-treated MCAO group compared with the sham-operated group (no MCAO) (Fig. 5F; *F*_2,20_ = 6.215, *p* < 0.01; 0.53 ± 0.09 (sham) vs. 4.43 ± 0.76 (water), *p* < 0.05; one-way ANOVA followed by Duncan post hoc test). Notably, the ME-treated MCAO group also had significantly less percentage of CD45^high^/CD11b^+^ cells compared with those in the water-treated MCAO group (Fig. 5F; 4.43 ± 0.76 (water) vs. 1.77 ± 0.51 (menthol) %, *p* < 0.05).

### Topically applied menthol did not improve post-ischemic lesion volumes or behavioral outcomes in *Trpm8*^−/−^ mice

**Fig. 6:**
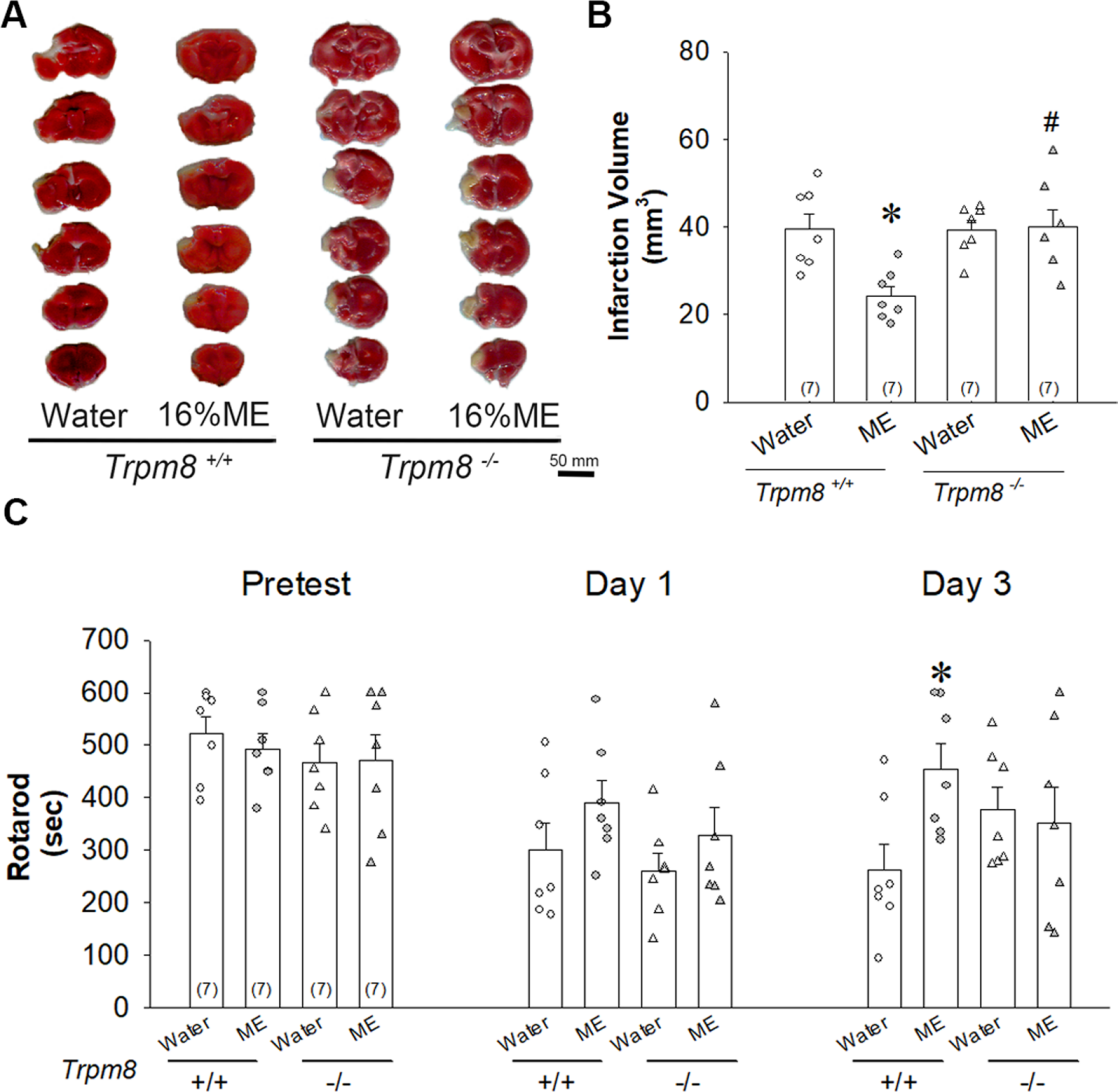
Topically applied menthol (ME) to paws does not attenuate MCAO injury in *Trpm8*^*−/−*^ mice. **A** TTC staining depicts the lesions 1 week after MCAO in *Trpm8*^+*/*+^ and *Trpm8*^*−/−*^ mice. The scale bar represents 5 mm. **B** The individual lesion volumes for water-or menthol-treated *Trpm8*^+*/*+^ and *Trpm8*^*−/−*^ mice are shown. **C** Rotarod performance of *Trpm8*^+*/*+^ and *Trpm8*^*−/−*^ mice. All data are expressed as the means ± S.E.M. *p < 0.05 vs water-treated *Trpm8*^+*/*+^ mice; ^#^*p* < 0.05 vs menthol-treated *Trpm8*^+*/*+^ mice. In each bar in **B**, **C**, the number of individuals of each group was shown within parentheses, and the symbols indicate the data of mice used in each study group

Figure 6A shows representative brain slices stained with TTC at 1 week after MCAO in *Trpm8*^+*/*+^ and *Trpm8*^*−/−*^ mice treated with water or menthol 16% applied to paws. TTC measurements revealed a 38.52% reduction in infarction volume in the *Trpm8*^+*/*+^ mice after menthol treatment (24.28 ± 2.15 mm^3^) compared with water treatment (39.49 ± 3.41 mm^3^; *p* < 0.05), whereas lesion volume was not improved in the menthol-treated *Trpm8*^*−/−*^ mice (40.15 ± 3.90 mm^3^) compared with water-treated *Trpm8*^+*/*+^ (39.50 ± 1.83 mm^3^) mice (*p* < 0.05). In addition, lesion volume was significant larger in the menthol-treated *Trpm8*^-/-^ mice (40.15 ± 3.90 mm^3^) compared with menthol-treated *Trpm8*^+/+^ mice (24.28 ± 2.15 mm^3^). (Fig. 6B; groups: *F*_1,24_ = 5.730, *p* < 0.05; genes: *F*_1,24_ = 6.821, *p* < 0.05; groups × genes: *F*_1,24_ = 7.110, *p* < 0.05; two-way ANOVA followed by Holm–Sidak post hoc test). Topically applied menthol in *Trpm8*^+/+^ mice significantly improved latency-to-fall in rotarod test (Fig. 6C; time points: *F*_2,36_ = 6.057, *p* < 0.01; groups: *F*_1,36_ = 4.250, *p* < 0.05; time points × groups: *F*_2,36_ = 2.675, *p* = 0.083; 261.4 ± 39.0 (water) vs. 455.0 ± 47.1 (menthol) on Day 3, *p* < 0.05; two-way ANOVA followed by Holm–Sidak post hoc test). Although the rotarod performance of *Trpm8*^*−/−*^ mice did not worsen compared to *Trpm8*^+/+^ mice after MCAO, topical menthol application to paws did not alter rotarod performance in *Trpm8*^*−/−*^ mice (Fig. 6C; time points: *F*_2,72_ = 11.947, *p* < 0.001; groups: *F*_1,72_ = 2.826, *p* = 0.097; genes: *F*_1,72_ = 1.388, *p* = 0.243; time points × groups: *F*_2,72_ = 0.960, *p* = 0.388; groups × genes: *F*_1,72_ = 1.273, *p* = 0.263; time points × genes: *F*_2,72_ = 0.654, *p* = 0.523; time points × groups × genes: *F*_2,72_ = 2.079, *p* = 0.132; three-way ANOVA followed by Holm–Sidak post hoc test).

## Discussion

**Fig. 7:**
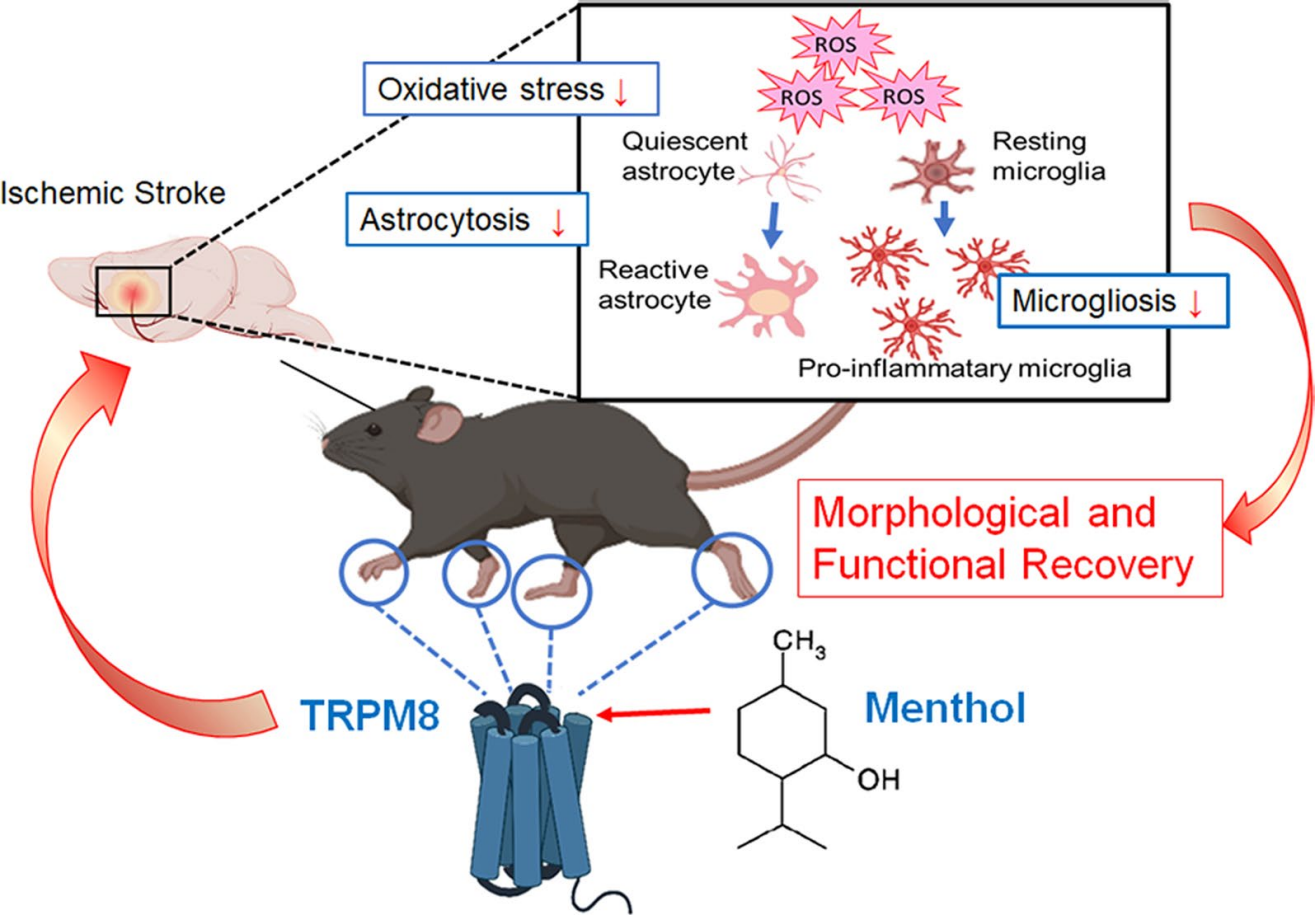
Schematic diagram of the effect of topically applied menthol to activate TRPM8 in the paws of mice against ischemic stroke. Topically applied menthol after 20-min MCAO reduces the oxidative stress in the infarcted cortex and decreases microgliosis and astrocytosis and consequently improves morphological and functional recovery

Our results reveal a previously unrecognized function of topically applied menthol in promoting functional recovery from MCAO damage. The protective effects of topically applied menthol were reversed by the TRPM8 antagonist, AMTB, and the local anesthetic, lidocaine. Since we observed that the benefits of topical menthol are associated with TRPM8 receptors in the peripheral nerve, it is to be expected that the abundant expression of TRPM8 in mouse paw skin was associated with significant improvements in infarction volume and behavior after MCAO injury by menthol application. Topically applied menthol reduced the expression of PARP and MDA content, but did not influence caspase-3 levels and apoptotic cells in the ipsilateral hemisphere. According to levels of GFAP, CD11b and Iba1 expression in *Trpm8*^+*/*+^ mice, topical menthol application to the paws suppressed the activation of astrocytes and microglia after focal cerebral ischemic injury. In *Trpm8*^*−/−*^ mice, topically applied menthol exacerbated the activation of astrocytes and microglia, and was not associated with any benefits in infarct volume or behavioral testing after MCAO injury. Our results show that topically applied menthol to mouse paws reduced oxidative stress and neuroinflammation and thus improved ischemic stroke via activation of the peripheral ion channel TRPM8. Figure 7 summarizes the effects of topically applied menthol to activate TRPM8 in the paws of mice against ischemic stroke.

We observed the effects of different dosages and routes of administration with menthol in the ischemic stroke model. Our data indicate more potent amelioration of stroke damage in mice when menthol is applied to their paws rather than their backs. IHC analyses revealed significantly higher TRPM8 expression in skin samples from the paws compared with skin samples from the backs. This result was reflected by significantly smaller infarct lesion volumes and superior sensorimotor function in mice treated with menthol 16% on the paws compared with on the back. These results also support the contention that TRPM8 expression in the paw skin is the main site of action for topically applied menthol to paws. Thus, even when skin from another part of the mouse body was indirectly moistened with menthol through touch contact, the very little amount of menthol had no protective effect against MCAO injury. Menthol concentration was important, as we observed significant therapeutic benefits with the dermal application of menthol 16% to the paws compared with menthol 8%. The concentration of menthol and the density of TRPM8 receptors were also important for neuroprotection of dermal menthol administration against cerebral ischemic injury. When administered to mice by oral gavage, menthol 200 mg/kg is associated with increased energy expenditure [38]. Moreover, oral menthol has shown anti-inflammatory effects in many different disease models [39–41]. However, in our study, oral menthol dosing did not assist with recovery from MCAO. These data indicate that topically applied menthol to the paws can improve infarction volume and sensorimotor dysfunction after ischemic stroke.

No research has as yet described the role of TRPM8 in stroke. Our results show that pretreatment with AMTB reversed the protective effects of menthol concerning infarction volume and behavioral deficits; these benefits associated with menthol were also abolished in *Trpm8*^*−/−*^ mice. The TRPM8 channel contributes to the cooling sensation of menthol, and cooling body temperature demonstrated therapeutic effectiveness after ischemic stroke [42]. TRPM8 is expressed on peripheral sensory nerve fibers as well as in hypothalamic and hindbrain nuclei in the CNS [43]. However, systemic hypothermia might affect all of organs and systems in the body [44]. Pharmacological activation of TRPM8 in peripheral nerve was realized with topical menthol application in our study. Researchers have speculated that TRPM8 may balance the proinflammatory responses of TRPV1 and TRPA1 during inflammation and thereby mediate chemosensory deactivation and inflammatory neuropeptide release [43]. TRPM8 activation inhibits mechanical allodynia and thermal hyperalgesia in chronic constriction injury-induced neuropathic pain after sciatic nerve ligation [45], while antagonism of TRPM8 downregulates TRPM8 protein levels and proinflammatory signaling, which thereby improves neuropathic pain [46]. It appears that TRPM8 plays a complex role in neuroprotection and neurological disorders.

In this study, activation of microglia and astrocytes after MCAO was suppressed by topically applied menthol. Recent study also showed activation of microglia was downregulated by hyperthermia after ischemic stroke [42]. In ischemic brains, microglia in the peri-infarct site phagocytose damaged and moribund cells within minutes of cerebral injury. If this process continues, it exacerbates the pathology of ischemia/reperfusion injury and proinflammatory cytokines are secreted, resulting in further damage [47]. Moreover, inflammatory stimuli induce production of reactive oxygen species (ROS) in damaged brain tissues after stroke [48, 49]. In cerebral ischemic injury, astrocytes are exposed to a broad spectrum of extracellular cues that result in astrogliosis and secretion of inflammatory mediators [49, 50], as well as upregulation of many potentially neurotoxic genes in the acute phase [51] and pronounced and long-lasting changes in astrogliosis producing scar formation [50, 51]. Treatments that can reduce astrogliosis and microgliosis promote recovery from ischemic stroke [52]. Our study results indicate that topically applied menthol shows promise in both aspects.

Following cerebral I/R injury, activated leukocytes infiltrate the infarcted zone [53, 54]. In this study, topical menthol application suppressed levels of CD11b protein expression. CD11b^+^ cells found within the brain parenchyma can express relatively high or low levels of CD45, a marker of all hematopoietic cells; high-level CD11b^+^ cells are infiltrating monocyte/macrophages, while low-level CD11b^+^ cells are resident microglia [30]. The accumulation of CD45^high^/CD11b^+^ cells previously reported in the brain parenchyma after MCAO [30, 37] was reduced by topical menthol treatment in our study. Our investigation of neutrophil expression in the infarcted brain revealed similar results (the data are not shown) to those of a previous report that found few parenchymal neutrophils in mice following acute MCAO [55].

Our results indicate that post-stroke topical application of menthol limits the infiltration of monocytes from the circulation.

Suppression of apoptosis and oxidation is typically regarded as a protective mechanism after ischemic injury. We observed that topical application of menthol to the paws reduced oxidative stress in the ischemic brain as reflected by decreased levels of MDA, a marker of lipid peroxidation. Increased PARP and caspase-3 expression in apoptosis is undesirable [56]. However, we found that topically applied menthol did not reduce caspase-3 expression, which was emphasized by the TUNEL assay results showing no decreases in apoptotic DNA fragmentation after topical menthol administration. Our findings resemble those of another experimental study that also recorded no change in levels of apoptosis after treating MCAO-induced stroke, with accompanying neuroprotective effects, decreased necrosis and improvements in neurological function [57]. We therefore speculate that topical menthol improves necrosis rather than affect the level of apoptosis, and thus reduces oxidative stress and infarct volume after MCAO. Another molecule increased with oxidative stress is the DNA repair enzyme PARP, which is normally activated by single-strand breaks during ischemic injury. PARP inhibition is cytoprotective in many disease models, so has been proposed for treatment of traumatic brain injury and stroke [58]. Study evidence has shown that treatment with a PARP inhibitor can protect human lens cells from oxidative stress-induced death [59] and attenuate neuronal cell death [60]. Moreover, PARP inhibition has been shown to downregulate the expression of inflammatory mediators and adhesion molecules in an in vitro model of the blood–brain barrier (BBB), enhancing barrier integrity and decreasing monocyte migration across the BBB models [61]. PARP inhibition has also shown neuroprotective effects by reducing cerebral infarction and BBB damage after cerebral ischemic injury [62]. These studies demonstrate how the suppression of oxidative stress and PARP are crucial for neuroprotection. We have shown that topically applied menthol uses these mechanisms to protect against ischemic stroke.

## Conclusions

Our study describes a unique, simple-to-use method for administering menthol treatment to the forepaws and hind paws of mice, which effectively ameliorated infarct volume and sensorimotor deficits in focal cerebral ischemic injury. We found that topically applied menthol was neuroprotective by decreasing PARP expression and oxidative stress, suppressing astrogliosis and microgliosis, and reducing the infiltration of monocytes and macrophages after MCAO. When applied to the skin of mouse paws, the neuroprotective effects of menthol were achieved via activation of TRPM8 receptors. As an adjuvant therapy for stroke, topical menthol application to the hands and feet could be an easy and convenient treatment for patients. This study provides new insights into the pathological processes that occur after acute ischemic stroke and a novel supplemental therapeutic strategy for stroke patients.

## Supplementary Information

**Additional file 1: Figure S1.** The method determined the infarct volume. The plots from TTC staining with the ipsilateral non-infarct area outlined in yellow and the ideally symmetrical ipsilateral and contralateral hemispheres outlined in green, all of which was mirrored on the ipsilateral side. Infarct area (mm^2^) = area of contralateral brain (green) - area of ipsilateral brain (yellow). Infarct volume (mm^3^) = [the summation of infarct areas (mm^2^) calculated on the front side and reverse side of each section] × 1 mm (the thick of each section)/2.

## Data Availability

The datasets used and/or analyzed during the current study are available from the corresponding author on reasonable request.

## Abbreviations

AMTB: N-(3-aminopropyl)-2-[(3-methylphenyl)methoxy]-N-(2-thienylmethyl) benzamide
CTCF: Corrected total cell fluorescence
DAB: 3,3’-Diaminobenzidine
GFAP: Glial fibrillary acidic protein
IBA1: Ionized calcium-binding adapter molecule 1
IHC: Immunohistochemistry
I/R: Ischemia–reperfusion
MCAO: Middle cerebral artery occlusion
MDA: Malondialdehyde
ME: DL-Menthol
PARP: Poly (ADP-ribose) polymerase
PBS: Phosphate buffered saline
TRPM8: Transient receptor potential melastatin 8
TTC: 2,3,5-Triphenyltetrazolium chloride
TUNEL: Terminal deoxynucleotidyl transferase dUTP nick-end labeling

## References

[1] Mehta A, Mahale R, Buddaraju K, Javali M, Acharya P, Srinivasa R (2019). Efficacy of neuroprotective drugs in acute ischemic stroke: is it helpful?. J Neurosci Rural Pract.

[2] Lin L, Wang X. Ischemia-reperfusion injury in the brain: mechanisms and potential therapeutic strategies. Biochem Pharmacol (Los Angel). 2016; 5. 10.4172/2167-0501.1000213 PMC5991620 29888120

[3] Warach S, Latour LL (2004). Evidence of reperfusion injury, exacerbated by thrombolytic therapy, in human focal brain ischemia using a novel imaging marker of early blood-brain barrier disruption. Stroke.

[4] Liu Y, Mikrani R, He Y, Faran Asraf Baig MM, Abbas M, Naveed M, Tang M, Zhang Q, Li C, Zhou X (2020). TRPM8 channels: a review of distribution and clinical role. Eur J Pharmacol.

[5] Zakharian E, Cao C, Rohacs T (2010). Gating of transient receptor potential melastatin 8 (TRPM8) channels activated by cold and chemical agonists in planar lipid bilayers. J Neurosci.

[6] Bautista DM, Siemens J, Glazer JM, Tsuruda PR, Basbaum AI, Stucky CL, Jordt SE, Julius D (2007). The menthol receptor TRPM8 is the principal detector of environmental cold. Nature.

[7] Colburn RW, Lubin ML, Stone DJ, Wang Y, Lawrence D, D'Andrea MR, Brandt MR, Liu Y, Flores CM, Qin N (2007). Attenuated cold sensitivity in TRPM8 null mice. Neuron.

[8] Dhaka A, Murray AN, Mathur J, Earley TJ, Petrus MJ, Patapoutian A (2007). TRPM8 is required for cold sensation in mice. Neuron.

[9] Peier AM, Moqrich A, Hergarden AC, Reeve AJ, Andersson DA, Story GM, Earley TJ, Dragoni I, McIntyre P, Bevan S, Patapoutian A (2002). A TRP channel that senses cold stimuli and menthol. Cell.

[10] McKemy DD, Neuhausser WM, Julius D (2002). Identification of a cold receptor reveals a general role for TRP channels in thermosensation. Nature.

[11] Beukema P, Cecil KL, Peterson E, Mann VR, Matsushita M, Takashima Y, Navlakha S, Barth AL (2018). TrpM8-mediated somatosensation in mouse neocortex. J Comp Neurol.

[12] Vay L, Gu C, McNaughton PA (2012). The thermo-TRP ion channel family: properties and therapeutic implications. Br J Pharmacol.

[13] Galeotti N, Di Cesare ML, Mazzanti G, Bartolini A, Ghelardini C (2002). Menthol: a natural analgesic compound. Neurosci Lett.

[14] Korting GW, Weigand U (1975). A new case of reticular hyperplasia connected with volatile oils. Hautarzt.

[15] Patel T, Ishiuji Y, Yosipovitch G (2007). Menthol: a refreshing look at this ancient compound. J Am Acad Dermatol.

[16] Liu B, Fan L, Balakrishna S, Sui A, Morris JB, Jordt SE (2013). TRPM8 is the principal mediator of menthol-induced analgesia of acute and inflammatory pain. Pain.

[17] Proudfoot CJ, Garry EM, Cottrell DF, Rosie R, Anderson H, Robertson DC, Fleetwood-Walker SM, Mitchell R (2006). Analgesia mediated by the TRPM8 cold receptor in chronic neuropathic pain. Curr Biol.

[18] Kardon AP, Polgar E, Hachisuka J, Snyder LM, Cameron D, Savage S, Cai X, Karnup S, Fan CR, Hemenway GM (2014). Dynorphin acts as a neuromodulator to inhibit itch in the dorsal horn of the spinal cord. Neuron.

[19] Chang LH, Lin HC, Huang SS, Chen IC, Chu KW, Chih CL, Liang YW, Lee YC, Chen YY, Lee YH, Lee IH (2018). Blockade of soluble epoxide hydrolase attenuates post-ischemic neuronal hyperexcitation and confers resilience against stroke with TrkB activation. Sci Rep.

[20] Kotoda M, Ishiyama T, Mitsui K, Hishiyama S, Matsukawa T (2017). Neuroprotective effects of amiodarone in a mouse model of ischemic stroke. BMC Anesthesiol.

[21] Hung SY, Liou HC, Kang KH, Wu RM, Wen CC, Fu WM (2008). Overexpression of heme oxygenase-1 protects dopaminergic neurons against 1-methyl-4-phenylpyridinium-induced neurotoxicity. Mol Pharmacol.

[22] Bouet V, Boulouard M, Toutain J, Divoux D, Bernaudin M, Schumann-Bard P, Freret T (2009). The adhesive removal test: a sensitive method to assess sensorimotor deficits in mice. Nat Protoc.

[23] Su HH, Liao JM, Wang YH, Chen KM, Lin CW, Lee IH, Li YJ, Huang JY, Tsai SK, Yen JC, Huang SS (2019). Exogenous GDF11 attenuates non-canonical TGF-beta signaling to protect the heart from acute myocardial ischemia-reperfusion injury. Basic Res Cardiol.

[24] Crowe A, Yue W. Semi-quantitative determination of protein expression using immunohistochemistry staining and analysis: an integrated protocol. BIO-PROTOCOL. 2019; 9. 10.21769/BioProtoc.3465 PMC6924920 31867411

[25] Jensen EC (2013). Quantitative analysis of histological staining and fluorescence using ImageJ. Anat Rec (Hoboken).

[26] Maidana DE, Tsoka P, Tian B, Dib B, Matsumoto H, Kataoka K, Lin H, Miller JW, Vavvas DG (2015). A novel ImageJ macro for automated cell death quantitation in the retina. Invest Ophthalmol Vis Sci.

[27] Lin JG, Lee YC, Tseng CH, Chen DY, Shih CY, MacDonald I, Hung SY, Chen YH (2016). Electroacupuncture inhibits pruritogen-induced spinal microglial activation in mice. Brain Res.

[28] Wang X, Johnson GA, Burghardt RC, Wu G, Bazer FW (2015). Uterine histotroph and conceptus development. I. Cooperative effects of arginine and secreted phosphoprotein 1 on proliferation of ovine trophectoderm cells via activation of the PDK1-Akt/PKB-TSC2-MTORC1 signaling cascade. Biol Reprod.

[29] Fitzpatrick M. Measuring cell fluorescence using ImageJ https://theolb.readthedocs.io/en/latest/imaging/measuring-cell-fluorescence-using-imagej.html#measuring-cell-fluorescence-using-imagej Accessed Sep 22, 2021.

[30] Ballesteros I, Cuartero MI, Moraga A, de la Parra J, Lizasoain I, Moro MA. Stereological and flow cytometry characterization of leukocyte subpopulations in models of transient or permanent cerebral ischemia. J Vis Exp 2014:e52031. 10.3791/52031 PMC4354492 25590380

[31] Martin E, El-Behi M, Fontaine B, Delarasse C. Analysis of microglia and monocyte-derived macrophages from the central nervous system by flow cytometry. JoVE 2017:e55781. 10.3791/55781 PMC5608497 28671658

[32] Var SR, Shetty AV, Grande AW, Low WC, Cheeran MC (2021). Microglia and macrophages in neuroprotection, neurogenesis, and emerging therapies for stroke. Cells.

[33] Deacon RM: Measuring motor coordination in mice. J Vis Exp 2013:e2609. 10.3791/2609 PMC3724562 23748408

[34] Barth TM, Jones TA, Schallert T (1990). Functional subdivisions of the rat somatic sensorimotor cortex. Behav Brain Res.

[35] Radak D, Katsiki N, Resanovic I, Jovanovic A, Sudar-Milovanovic E, Zafirovic S, Mousad SA, Isenovic ER (2017). Apoptosis and acute brain ischemia in ischemic stroke. Curr Vasc Pharmacol.

[36] Li Y, Wang Y, Yao Y, Griffiths BB, Feng L, Tao T, Wang F, Xu B, Stary CM, Zhao H (2020). Systematic study of the immune components after ischemic stroke using CyTOF techniques. J Immunol Res.

[37] Inacio AR, Liu Y, Clausen BH, Svensson M, Kucharz K, Yang Y, Stankovich T, Khorooshi R, Lambertsen KL, Issazadeh-Navikas S, Deierborg T (2015). Endogenous IFN-beta signaling exerts anti-inflammatory actions in experimentally induced focal cerebral ischemia. J Neuroinflammation.

[38] Khare P, Mangal P, Baboota RK, Jagtap S, Kumar V, Singh DP, Boparai RK, Sharma SS, Khardori R, Bhadada SK (2018). Involvement of glucagon in preventive effect of menthol against high fat diet induced obesity in mice. Front Pharmacol.

[39] Du J, Liu D, Zhang X, Zhou A, Su Y, He D, Fu S, Gao F (2020). Menthol protects dopaminergic neurons against inflammation-mediated damage in lipopolysaccharide (LPS)-Evoked model of Parkinson’s disease. Int Immunopharmacol.

[40] Wang Q, Yang Y, Chen K, Li D, Tang B, Peng K, Wang Z, Yang P, Yang D, Yang Y (2020). Dietary menthol attenuates inflammation and cardiac remodeling after myocardial infarction via the transient receptor potential melastatin 8. Am J Hypertens.

[41] Feitosa KA, Zaia MG, Rodrigues V, Castro CA, Correia RO, Pinto FG, Rossi K, Avo LRS, Afonso A, Anibal FF (2017). Menthol and menthone associated with acetylsalicylic acid and their relation to the hepatic fibrosis in *Schistosoma mansoni* infected mice. Front Pharmacol.

[42] Kim JY, Kim JH, Park J, Beom JH, Chung SP, You JS, Lee JE (2021). Targeted temperature management at 36 degrees C shows therapeutic effectiveness via alteration of microglial activation and polarization after ischemic stroke. Transl Stroke Res.

[43] Silverman HA, Chen A, Kravatz NL, Chavan SS, Chang EH (2020). Involvement of neural transient receptor potential channels in peripheral inflammation. Front Immunol.

[44] Nielsen N, Wetterslev J, Cronberg T, Erlinge D, Gasche Y, Hassager C, Horn J, Hovdenes J, Kjaergaard J, Kuiper M (2013). Targeted temperature management at 33 degrees C versus 36 degrees C after cardiac arrest. N Engl J Med.

[45] Su L, Wang C, Yu YH, Ren YY, Xie KL, Wang GL (2011). Role of TRPM8 in dorsal root ganglion in nerve injury-induced chronic pain. BMC Neurosci.

[46] Cao S, Li Q, Hou J, Li Z, Cao X, Liu X, Qin B (2019). Intrathecal TRPM8 blocking attenuates cold hyperalgesia via PKC and NF-κB signaling in the dorsal root ganglion of rats with neuropathic pain. J Pain Res.

[47] Takeda H, Yamaguchi T, Yano H, Tanaka J (2021). Microglial metabolic disturbances and neuroinflammation in cerebral infarction. J Pharmacol Sci.

[48] McCann SK, Roulston CL (2013). NADPH oxidase as a therapeutic target for neuroprotection against ischaemic stroke: future perspectives. Brain Sci.

[49] Sun L, Zhang Y, Liu E, Ma Q, Anatol M, Han H, Yan J (2019). The roles of astrocyte in the brain pathologies following ischemic stroke. Brain Inj.

[50] Sofroniew MV (2014). Astrogliosis. Cold Spring Harb Perspect Biol.

[51] Rakers C, Schleif M, Blank N, Matuskova H, Ulas T, Handler K, Torres SV, Schumacher T, Tai K, Schultze JL (2019). Stroke target identification guided by astrocyte transcriptome analysis. Glia.

[52] Sims NR, Yew WP (2017). Reactive astrogliosis in stroke: contributions of astrocytes to recovery of neurological function. Neurochem Int.

[53] Beller E, Reuter L, Kluge A, Preibisch C, Lindauer U, Bogdanov A, Lammer F, Delbridge C, Matiasek K, Schwaiger BJ (2018). Pilot study to assess visualization and therapy of inflammatory mechanisms after vessel reopening in a mouse stroke model. Sci Rep.

[54] Wang N, Fei C, Chu F, Huang S, Pan L, Peng D, Duan X. Taohong Siwu decoction regulates cell necrosis and neuroinflammation in the rat middle cerebral artery occlusion model. Front Pharmacol. 2021; 12. 10.3389/fphar.2021.732358 PMC8384077 34447315

[55] Otxoa-De-Amezaga A, Gallizioli M, Pedragosa J, Justicia C, Miró-Mur F, Salas-Perdomo A, Díaz-Marugan L, Gunzer M, Planas AM (2019). Location of neutrophils in different compartments of the damaged mouse brain after severe ischemia/reperfusion. Stroke.

[56] Zhang F, Lau SS, Monks TJ (2012). A dual role for poly(ADP-ribose) polymerase-1 during caspase-dependent apoptosis. Toxicol Sci.

[57] Di Y, He YL, Zhao T, Huang X, Wu KW, Liu SH, Zhao YQ, Fan M, Wu LY, Zhu LL (2015). Methylene blue reduces acute cerebral ischemic injury via the induction of mitophagy. Mol Med.

[58] Berger NA, Besson VC, Boulares AH, Burkle A, Chiarugi A, Clark RS, Curtin NJ, Cuzzocrea S, Dawson TM, Dawson VL (2018). Opportunities for the repurposing of PARP inhibitors for the therapy of non-oncological diseases. Br J Pharmacol.

[59] Smith AJ, Ball SS, Bowater RP, Wormstone IM (2016). PARP-1 inhibition influences the oxidative stress response of the human lens. Redox Biol.

[60] Xu JC, Fan J, Wang X, Eacker SM, Kam TI, Chen L, Yin X, Zhu J, Chi Z, Jiang H (2016). Cultured networks of excitatory projection neurons and inhibitory interneurons for studying human cortical neurotoxicity. Sci Transl Med.

[61] Rom S, Zuluaga-Ramirez V, Dykstra H, Reichenbach NL, Ramirez SH, Persidsky Y (2015). Poly(ADP-ribose) polymerase-1 inhibition in brain endothelium protects the blood-brain barrier under physiologic and neuroinflammatory conditions. J Cereb Blood Flow Metab.

[62] Teng F, Zhu L, Su J, Zhang X, Li N, Nie Z, Jin L (2016). Neuroprotective effects of Poly(ADP-ribose)polymerase inhibitor olaparib in transient cerebral ischemia. Neurochem Res.

