# Supplementary material for: Activation of peripheral TRPM8 mitigates ischemic stroke by topically applied menthol
Source: J Neuroinflammation. 2022 Jul 27;19:192. doi: 10.1186/s12974-022-02553-4 (PMC9327358; doi:10.1186/s12974-022-02553-4)
Supplement: Supplementary file 1 — Additional file 1: Figure S1. The method determined the infarct volume. The plots from TTC staining with the ipsilateral non-infarct area outlined in yellow and the ideally symmetrical ipsilateral and contralateral hemispheres outlined in green, all of which was mirrored on the ipsilateral side. Infarct area (mm2) = area of contralateral brain (green) - area of ipsilateral brain (yellow). Infarct volume (mm3) = [the summation of infarct areas (mm2) calculated on the front side and reverse side of each section] × 1 mm (the thick of each section)/2. [file 12974_2022_2553_MOESM1_ESM.docx]

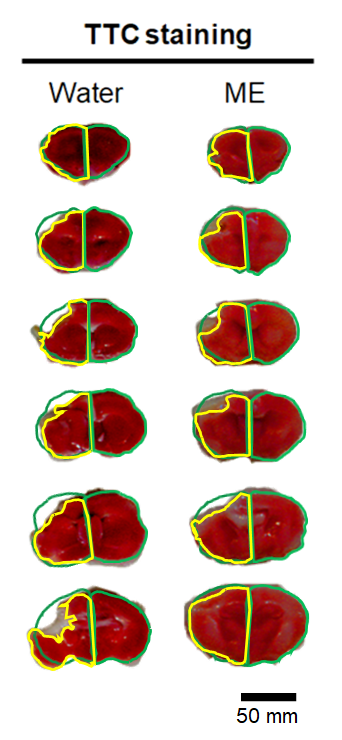


**Fig S1. The method determined the infarct volume.** The plots from TTC staining with the ipsilateral non-infarct area outlined in yellow and the ideally symmetrical ipsilateral and contralateral hemispheres outlined in green, all of which was mirrored on the ipsilateral side. Infarct area (mm^2^) = area of contralateral brain (green) - area of ipsilateral brain (yellow). Infarct volume (mm^3^) = [the summation of infarct areas (mm^2^) calculated on the front side and reverse side of each section] × 1 mm (the thick of each section)/2.
